# Prevalences and associated factors of electrocardiographic abnormalities in Chinese adults: a cross-sectional study

**DOI:** 10.1186/s12872-020-01698-5

**Published:** 2020-09-11

**Authors:** Liping Yu, Xiaojun Ye, Zhaojun Yang, Wenying Yang, Bo Zhang, on behalf of the China National Diabetes and Metabolic Disorders Study Group

**Affiliations:** 1 Department of Endocrinology, China-Japan Friendship Hospital, Beijing, China; 2 Department of Cardiology, China-Japan Friendship Hospital, Beijing, China

**Keywords:** Prevalence, Electrocardiogram abnormalities, Arrhythmia, CVD risk factors

## Abstract

**Background:**

Electrocardiogram (ECG) is widely used to screen cardiac diseases. To date, no large population study has provided estimates of the prevalences of ECG findings in China. We aim to investigate the prevalences and associated factors of ECG abnormalities in a general population of Chinese adults.

**Methods:**

ECG data were obtained from 34,965 participants in the 2007–2008 China National Diabetes and Metabolic Disorders Study. ECG abnormalities were classified according to the Minnesota coding (MC) criteria. Prevalences of variant ECG abnormalities were calculated. The associations between ECG abnormalities and gender, age and other risk factors for cardiovascular diseases (CVD) were analyzed by multivariate logistic regression test.

**Results:**

The prevalences of major arrhythmias were 1.70, 2.37 and 1.04% in the whole population, men and women, respectively. Atrial fibrillation/flutter was found in 0.35% of men and 0.20% of women. ST depression and T abnormalities accounted for 10.96, 7.54 and 14.32% in the whole population, men and women, respectively. Independent of gender and other CVD risk factors, older age significantly increased the odds of having atrial fibrillation/flutter, complete left bundle branch block, complete right bundle branch block, sinus tachycardia, atrial/junctional/ventricular premature beats, ST depression and T abnormalities, tall R wave left, left/right atrial hypertrophy, left axis deviation and low voltage. Hypertension, overweight, obesity and hypercholesterolemia all independently increased the odds of having ST depression and T abnormalities. History of cardiovascular/cerebrovascular diseases was positively associated with major arrhythmias, ST depression and T abnormalities and tall R wave left.

**Conclusions:**

This study provides estimates of the prevalences of ECG findings in a large population of Chinese adults. Gender, age, CVD risk factors and history of cardiovascular/cerebrovascular diseases were significantly associated with ECG abnormalities.

## Background

The electrocardiogram (ECG) is an inexpensive and convenient tool that has been widely used to screen arrhythmias and cardiovascular diseases (CVDs). The prevalences of variant ECG abnormalities and their association with age and CVD risk factors have been reported in several large population studies conducted in North American, South American and European countries [1–3]. The prevalence of major ECG abnormalities has been reported to vary from 6.0 to 11.3% in men and from 4.3 to 12.9% in women in previous studies, with differences dependent on various racial backgrounds and targeted age groups [1–4]. Older age has been positively associated with the prevalences of major ECG abnormalities and several specific ECG findings [2, 3, 5]. A previous cross-sectional survey reported that arrhythmias accounted for 4.8% in men and 3.6% in women from 65 to 74 years old, while the prevalences of arrhythmias were less than 0.5% in both men and women under 45 years old [1]. Other CVD risk factors, such as hypertension and diabetes, increased the odds of having major ECG abnormalities and some specific ECG findings [1, 2].

To date, no large population study has provided estimates of the prevalences of ECG findings in different age groups in China. Most previous ECG studies performed in China were regional or focused on specific types of ECG findings [6, 7]. The ECG data presented in this study were obtained from 34,965 participants in the China National Diabetes and Metabolic Disorders Study from June 2007 to May 2008. The objective of the present study was to investigate the prevalences of ECG findings in the general population of Chinese adults and determine the associations between ECG abnormalities and age, gender and other CVD risk factors.

## Methods

### Participants studied

The China National Diabetes and Metabolic Disorders Study was a population-based cross-sectional study carried out from June 2007 to May 2008 [8]. The details of its sampling methods have been described previously [8, 9]. Briefly, 47,325 participants (18,976 men and 28,349 women aged ≥20 years old) from 152 urban street districts and 112 rural villages in 14 provinces completed the study [8, 9]. First, 1086 persons were excluded due to the missing of demographic information or glucose level data. Second, ECG data from some subcenters (including all subcenters from Beijing, two subcenters from Hunan, two subcenters from Jiangsu and one subcenter from Xinjiang) were not well recorded; thus, data from these subcenters (data from 10,951 persons) were excluded from the analysis. Third, 100 persons missed smoking history, 93 persons missed BMI records, while 141 persons missed lipid data. Thus, 323 persons were excluded due to the missing of smoking history records, BMI records or lipid data. Ultimately, 34,965 participants (13,983 males and 20,982 females) were included in this analysis. This study was approved by the institutional review board and the ethics committee of local institutions [8].

### Study-outcome definitions

The design and methods of the China National Diabetes and Metabolic Disorders Study were reported previously [8, 9]. Briefly speaking, interviews were conducted and standard questionnaires were completed to obtain the information about demographical characteristics, lifestyle risk factors, personal medical history, treatment of diseases and family disease history. Fasting blood samples were collected from the participants to test liver function, renal function and lipid levels. ECGs and measurements of blood pressure, waist circumference, height and weight were conducted for participants after an overnight fast by well-trained clinical staffs. Participants then received a standard oral glucose tolerance test. Diabetes mellitus was defined as fasting plasma glucose ≥7.00 mmol/L, 2-h plasma glucose ≥11.10 mmol/L or using glucose-lowering medications with a history of diabetes. Prediabetes was defined as fasting plasma glucose 6.10–6.99 mmol/L or 2-h plasma glucose 7.80–11.09 mmol/L without any evidence of diagnosis of diabetes. Hypercholesterolemia was defined as total cholesterol ≥6.22 mmol/L, LDL cholesterol ≥4.14 mmol/L or using cholesterol-lowering medications with a history of hypercholesterolemia. Hypertension was defined as systolic blood pressure ≥ 140 mmHg, diastolic blood pressure ≥ 90 mmHg or using antihypertensive medications with a history of hypertension. Obesity was defined as BMI ≥28 kg/m^2^, while overweight was defined as BMI 24–27.9 kg/m^2^ according to the criteria adopted by the Chinese Society of Endocrinology. The definition of smoking history was having smoked at least 100 cigarettes in the past. Rural areas referred to rural villages. Urban areas referred to city districts. The selection of geographic regions was described in a previous article [8].

Twelve-lead ECGs were conducted with the subject in the supine position. ECGs were read and recorded by two trained physicians in each subcenter. ECG data were classified based on the Minnesota coding (MC) criteria [1, 2, 5]. Major arrhythmias included atrial fibrillation or flutter (MC 8–3), complete left bundle branch block (LBBB, MC 7–1), complete right bundle branch block (RBBB, MC 7–2), nonspecific intraventricular conduction delay (IVCD, MC 7–4), Mobitz Type II or III atrial-ventricular (AV) conduction defects (MC 6–1, 6–2), supraventricular or ventricular rhythm/tachycardia (MC 8–4-1, 8–4-2, 8–2-2, 8–2-3), Wolff-Parkinson-White (WPW, MC 6–4) and artificial pacemaker (MC 6–8). Minor arrhythmias mainly included sinus bradycardia (MC 8–8), sinus tachycardia (MC 8–7), atrial or junctional or ventricular premature beats (MC 8–1-1, 8–1-2, 8–1-3), incomplete RBBB (MC 7–3), Mobitz Type I AV conduction defect (MC 6–3) and short PR interval (MC 6–5). Other types of ECG abnormalities were classified and analyzed as well, including ST depression and T abnormalities (MC 4–1, 4–2, 4–3, 4–4, 5–1, 5–2, 5–3 or 5–4), Q wave abnormalities (MC 1–1, 1–2), Q wave abnormalities plus ischemic ST-T abnormalities (MC ‘1-1 or 1-2’ plus ‘4-1, 4-2, 4-3, 4-4, 5-1, 5-2, 5-3 or 5-4’), ST elevation (MC 9–2), tall R wave left (MC 3–1 or 3–3), tall R wave right (MC 3–2), left/right atrial hypertrophy (MC 9–3 or 9–6), left axis deviation (MC 2–1), right axis deviation (MC 2–2) and low voltage (MC 9–1).

### Statistical methods

The prevalence calculation and significance evaluation, performed using SUDAAN software (version 10, Research Triangle Institute) in this study, were weighted to represent the population of Chinese adults (≥20 years old) based on the Chinese population distribution data in 2006 [8]. The age- and gender- standardized prevalences of ECG abnormalities were calculated for the whole population, for males and females, and for different age groups. Multivariate logistic regression analysis was conducted using SUDAAN software (version 10, Research Triangle Institute) to investigate associations of gender, age, metabolic factors, smoking history and rural/urban areas with the odds of ECG abnormalities. Factors simultaneously adjusted to calculate the odds ratios included gender, age classes, smoking history, hypertension, blood glucose classes, BMI classes, hypercholesterolemia and rural/urban areas.

## Results

This study included 34,965 participants (13,983 men and 20,982 women). The baseline characteristics of the participants are summarized in Table 1. The mean ages of men and women were both 44.8 years old. There were no significant differences comparing the mean age (*p* = 0.765) and the age classes (*p* = 0.886) between men and women. Men had significantly higher prevalences of overweight, obesity, presence of smoking history, hypertension, diabetes and presence of one or more CVD risk factors (Table 1).

**Table 1:** Characteristics of the 34,965 participants in this study

Characteristics	Men (*n* = 13,983)	Women (*n* = 20,982)	*P* values^#^
Age, y [mean (95% CI)]	44.8 (44.7–44.9)	44.8 (44.7–44.8)	0.765
Age classes			0.886
20–29 years old, %	17.2	17.7	0.396
30–39 years old, %	23.8	24.0	0.738
40–49 years old, %	22.5	22.3	0.777
50–59 years old, %	18.6	17.9	0.245
60–69 years old, %	10.5	9.8	0.123
≥ 70 years old, %	7.5	8.3	0.310
BMI, kg/m^2^ [mean (95% CI)]	24.1 (24.0–24.2)	23.5 (23.4–23.5)	< 0.001
Overweight, %	34.9	29.4	< 0.001
Obesity, %	14.0	10.8	< 0.001
Smoking, %	58.2	3.6	< 0.001
Hypertension, %	31.5	25.3	< 0.001
Prediabetes, %	16.2	15.4	0.244
Diabetes, %	10.9	8.9	< 0.001
FPG, mmol/L [mean (95% CI)]	5.4 (5.3–5.4)	5.3 (5.2–5.3)	0.073
PG2h, mmol/L [mean (95% CI)]	6.9 (6.8–7.0)	7.0 (6.9–7.1)	0.328
Hypercholesterolemia, %	11.1	10.5	0.244
Rural area, %	47.7	47.6	0.955
Urban area, %	52.3	52.4	0.955
With cardiovascular/cerebrovascular diseases, %	3.1	2.5	0.121
CVD risk groups			< 0.001
Without CVD risk factors, %	22.2	60.2	< 0.001
With one CVD risk factor, %	43.9	25.1	< 0.001
With two CVD risk factors, %	22.5	10.8	< 0.001
With three or more CVD risk factors, %	11.3	4.0	< 0.001

The percentages shown above were compared by Chi-square test. ^#^ The underlined numbers indicated the significant differences (p < 0.05). The quantitative values of age, BMI, FPG and PG2h were indicated as mean (95% CI) and compared by two-tailed t test. CVD risk factors include hypertension, diabetes, obesity, hypercholesterolemia and smoking history. Older age was not included in the CVD risk factors for analysis because age was mentioned and analyzed separately. *BMI* body mass index, *FPG* fasting plasma glucose level, *PG2h* plasma glucose level of 2 h after oral glucose tolerance test, *CVD* cardiovascular disease, *CI* confidence interval

The weighted prevalences of arrhythmias in men, women and all participants with different ages are summarized in Table 2. Major arrhythmias accounted for 1.70% of all participants. The weighted prevalence of major arrhythmias in men was higher than in women (2.37% vs 1.04% in men vs women) (Table 2). The weighted prevalences of major arrhythmias in the young (20–44 years old), middle (45–59 years old) and older age (≥60 years old) groups were 1.30, 2.37 and 5.56% in men, and 0.58, 1.06 and 2.38% in women, respectively. Specifically, atrial fibrillation/flutter (MC 8–3) accounted for 0.28, 0.35 and 0.20% in the whole population, men and women respectively. Complete RBBB (MC 7–2) had the highest prevalence among all major arrhythmias, with the weighted prevalence of 0.85, 1.16 and 0.55% in the whole population, men and women respectively. Minor arrhythmias accounted for 9.92% in all participants. In the young, middle and older age groups, the weighted prevalences of minor arrhythmia were 11.05, 10.82 and 14.26% in men, and 6.58, 7.85 and 14.17% in women, respectively. Individuals in the older age group had higher prevalences of atrial fibrillation/flutter, complete RBBB and atrial/junctional/ventricular premature beats in both men and women, compared with those in the young age group. In addition, gender had an important impact on some specific types of arrhythmia. Complete LBBB, complete RBBB, nonspecific IVCD, sinus bradycardia, incomplete RBBB and Mobitz type I AV conduction defect were more common in men than in women, while sinus tachycardia was more frequent in women than in men (Table 2).

**Table 2:** Prevalences of arrhythmias in men, women and all participants

ECG abnormalities	All participants, No. (percentage)	Men (n = 13,983)				Women (n = 20,982)				P value^**#**^ (men vs women)
		20-44y	45-59y	≥60y	All ages	20-44y	45-59y	≥60y	All ages	
**Major arrhythmias**	**550 (1.70%)**	**1.30%**	**2.37%**	5.56%***	**2.37%**	**0.58%**	**1.06%**	2.38%***	**1.04%**	**< 0.001**
Atrial fibrillation or flutter	77 (0.28%)	0.14%	0.23%	1.20%**	0.35%	0.08%	0.05%	0.80%*	0.20%	0.130
Complete LBBB	28 (0.12%)	0.06%	0.20%	0.61%	0.20%	0.01%	0.03%	0.15%*	0.05%	0.037
Complete RBBB	289 (0.85%)	0.51%	1.21%	2.97%***	1.16%	0.35%	0.59%	1.10%**	0.55%	0.001
Nonspecific IVCD	59 (0.20%)	0.29%	0.43%	0.59%	0.38%	0.00%	0.06%	0.03%	0.02%	< 0.001
Mobitz Type II or III AV conduction defects	16 (0.04%)	0.01%	0.07%	0.04%	0.03%	0.00%	0.17%	0.02%	0.05%	0.536
Supraventricular or ventricular rhythm/tachycardia	28 (0.09%)	0.14%	0.14%	0.12%	0.14%	0.01%	0.10%	0.07%	0.05%	0.084
WPW	44 (0.09%)	0.14%	0.04%	0.02%**	0.09%	0.12%	0.04%	0.05%	0.09%	0.926
Artificial pacemaker	9 (0.03%)	0.01%	0.04%	0.00%	0.02%	0.00%	0.01%	0.16%	0.03%	0.117
**Minor arrhythmias**	**3046 (9.92%)**	**11.05%**	**10.82%**	14.26%*	**11.58%**	**6.58%**	**7.85%**	14.17%***	**8.29%**	< 0.001
Sinus bradycardia	703 (2.81%)	3.99%	4.29%	3.22%	3.94%	1.62%	2.03%	1.39%	1.70%	< 0.001
Sinus tachycardia	540 (1.68%)	0.94%	1.07%	2.06%*	1.18%	1.78%	1.38%	4.52%	2.14%	0.004
Atrial or junctional or ventricular premature beats	544 (1.57%)	0.91%	1.04%	4.33%***	1.58%	0.87%	1.73%**	3.41%***	1.57%	0.945
Incomplete RBBB	285 (0.97%)	1.53%	1.47%	0.94%	1.40%	0.34%	0.85%*	0.67%	0.55%	< 0.001
Mobitz Type I AV conduction defect	141 (0.47%)	0.43%	0.85%*	0.79%	0.62%	0.15%	0.17%	1.08%	0.32%	0.045
Short PR interval	241 (0.70%)	0.84%	0.26%*	0.13%*	0.54%	1.02%	0.89%	0.34%***	0.86%	0.084
Other minor arrhythmias	592 (1.73%)	2.40%	1.84%	2.80%	2.31%	0.81%	0.79%	2.75%	1.15%	< 0.001

The percentages shown above were compared by Chi-square or Fisher’s test. *, * and *** indicated the *p* values comparing the percentages of ECG findings in the middle/older age group with those in the young age group (* p < 0.05, ** p < 0.01, *** p < 0.001). ^#^ The *p* values revealed the differences comparing the percentages of ECG findings between men and women. The underlined *p* values indicated the significant differences (p < 0.05). *LBBB* left bundle branch block, *RBBB* right bundle branch block, *IVCD* intravascular conducting delay, *AV* atrial-ventricular, *WPW* Wolff-Parkinson-White

With respect to other ECG abnormalities except arrhythmias, ST depression and T abnormalities and tall R wave left had higher prevalences than other specific ECG types (Table 3). The ST depression and T abnormalities accounted for 10.96, 7.54 and 14.32% in the whole population, men and women respectively. Tall R wave left accounted for 4.42, 5.83 and 3.05% in the whole population, men and women respectively. Participants in the older group had higher prevalences of ST depression and T abnormalities, tall R wave left and left axis deviation compared with those in the young group. Gender also had influence on these ECG abnormalities. Compared with women, men had significantly higher prevalences of Q wave abnormalities, ST elevation, tall R wave left, left axis deviation and right axis deviation. Women had higher prevalences of ST depression and T abnormalities and low voltage compared with men (Table 3).

**Table 3:** Prevalences of other ECG abnormalities (except arrhythmias) in men, women and all participants

ECG abnormalities	All participants, No. (percentage)	Men (n = 13,983)				Women (n = 20,982)				P value^**#**^ (men vs women)
		20-44y	45-59y	≥60y	All ages	20-44y	45-59y	≥60y	All ages	
ST depression and T abnormalities	4192 (10.96%)	5.26%	9.30%***	11.44%***	7.54%	8.36%	18.46%***	25.19%***	14.32%	< 0.001
Q wave abnormalities	416 (1.28%)	1.35%	1.77%	1.86%	1.55%	0.78%	1.23%	1.31%	1.00%	0.003
Q wave abnormalities plus ST-T ischemic abnormalities	75 (0.16%)	0.14%	0.05%	0.55%	0.19%	0.07%	0.17%	0.30%*	0.14%	0.414
ST elevation	239 (0.92%)	2.81%	0.88%***	0.20%***	1.78%	0.06%	0.16%	0.02%	0.08%	< 0.001
Tall R wave left	1042 (4.42%)	4.94%	7.16%*	6.28%	5.83%	1.21%	3.40%***	7.89%***	3.05%	< 0.001
Tall R wave right	54 (0.22%)	0.23%	0.13%	0.41%	0.23%	0.28%	0.06%	0.22%	0.20%	0.765
Left/right atrial hypertrophy	64 (0.31%)	0.21%	0.43%	1.05%	0.43%	0.06%	0.27%*	0.52%*	0.21%	0.063
Left axis deviation	698 (2.13%)	1.41%	3.31%***	4.36%***	2.48%	0.98%	2.22%***	3.35%***	1.78%	0.003
Right axis deviation	254 (0.67%)	0.99%	0.72%	0.80%	0.88%	0.67%	0.31%*	0.15%***	0.47%	0.008
Low voltage	325 (0.97%)	0.45%	0.81%	1.16%	0.68%	1.14%	1.18%	1.67%	1.24%	0.001

The percentages shown above were compared by Chi-square or Fisher’s test. *, * and *** indicated the p values comparing the percentages of ECG findings in the middle/older age group with those in the young age group (* p < 0.05, ** p < 0.01, *** p < 0.001). ^#^ The p values revealed the differences comparing the percentages of ECG findings between men and women. The underlined p values indicated the significant differences (p < 0.05)

To identify the factors that influence each arrhythmia ECG type, multivariate logistic regression analysis was conducted, and the results are displayed in Table 4. Male gender, older age and living in rural area were significantly associated with major arrhythmias. Older age (at least 60 years old) significantly increased the odds of having atrial fibrillation/flutter, complete LBBB, complete RBBB, nonspecific IVCD, sinus tachycardia, atrial/junctional/ventricular premature beats and Mobitz Type I AV conduction defect. Smoking was positively associated with supraventricular or ventricular rhythm/tachycardia and incomplete RBBB. Hypertension increased the odds of having sinus tachycardia and Mobitz Type I AV conduction defect. Diabetes, obesity and hypercholesterolemia were not positively associated with any arrhythmia ECG type. Residents living in rural area had higher odds of obtaining complete LBBB, nonspecific IVCD, sinus bradycardia and incomplete RBBB compared to those living in urban areas (Table 4).

**Table 4:** The odds ratios of the effects of multiple factors on arrhythmias

ECG abnormalities	Gender (male vs female)	45–59 years old	≥60 years old	Smoking	Hypertension	Diabetes	Obesity	Hyper-cholesterolemia	Rural (vs urban)
**Major arrhythmias**	**2.14** **(1.56–2.95)**	**1.83** **(1.29–2.58)**	**4.90** **(3.48–6.92)**	**1.22** **(0.89–1.69)**	**1.29** **(0.95–1.77)**	**0.86** **(0.57–1.29)**	**1.20** **(0.77–1.87)**	**1.37** **(0.93–2.04)**	**1.39** **(1.06–1.81)**
Atrial fibrillation/flutter	1.91 (0.83–4.42)	1.23 (0.37–4.05)	9.39 (3.63–24.29)	1.01 (0.42–2.46)	1.64 (0.74–3.65)	0.68 (0.25–1.82)	0.72 (0.19–2.69)	2.21 (0.86–5.68)	1.91 (0.95–3.86)
Complete LBBB	3.06 (0.77–12.19)	2.84 (0.53–15.07)	11.08 (2.43–50.49)	2.10 (0.53–8.31)	0.95 (0.33–2.72)	1.57 (0.25–9.94)	0.98 (0.30–3.15)	2.41 (0.61–9.50)	3.72 (1.25–11.06)
Complete RBBB	1.92 (1.23–3.01)	2.04 (1.22–3.41)	5.27 (3.19–8.70)	1.23 (0.78–1.94)	1.36 (0.87–2.13)	1.07 (0.66–1.74)	1.45 (0.76–2.77)	1.22 (0.70–2.11)	1.02 (0.69–1.50)
Nonspecific IVCD	18.43 (5.44–62.44)	1.88 (0.79–4.47)	2.89 (1.03–8.09)	1.04 (0.47–2.33)	1.02 (0.41–2.50)	0.23 (0.05–1.02)	0.95 (0.25–3.59)	1.25 (0.40–3.92)	2.53 (1.29–4.96)
Mobitz Type II or III AV conduction defects	0.96 (0.26–3.58)	12.09 (3.11–47.04)	2.74 (0.49–15.26)	0.55 (0.13–2.34)	2.88 (0.58–14.32)	0.69 (0.13–3.62)	1.14 (0.12–10.88)	0.54 (0.12–2.38)	1.62 (0.47–5.59)
Supraventricular or ventricular rhythm/tachycardia	1.40 (0.54–3.65)	1.69 (0.54–5.30)	1.66 (0.45–6.17)	3.23 (1.22–8.57)	1.60 (0.50–5.12)	0.53 (0.12–2.42)	0.70 (0.18–2.80)	0.56 (0.14–2.16)	1.20 (0.41–3.53)
WPW	1.27 (0.55–2.93)	0.46 (0.16–1.36)	0.58 (0.12–2.77)	0.75 (0.28–2.00)	0.31 (0.10–1.00)	0.39 (0.10–1.44)	2.50 (0.67–9.33)	0.74 (0.16–3.36)	1.24 (0.58–2.65)
**Minor arrhythmias**	**1.34** **(1.13–1.59)**	**1.19** **(1.03–1.38)**	**2.08** **(1.69–2.57)**	**1.20** **(1.02–1.41)**	**1.29** **(1.08–1.52)**	**0.99** **(0.77–1.28)**	**0.62** **(0.50–0.77)**	**0.82** **(0.63–1.07)**	**1.19** **(1.04–1.37)**
Sinus bradycardia	2.21 (1.66–2.93)	1.47 (1.13–1.90)	1.41 (0.97–2.04)	1.29 (0.97–1.72)	0.71 (0.53–0.96)	0.65 (0.42–1.00)	0.67 (0.44–1.01)	0.64 (0.42–0.97)	2.03 (1.64–2.50)
Sinus tachycardia	0.64 (0.40–1.02)	0.84 (0.59–1.20)	2.10 (1.15–3.83)	0.75 (0.46–1.22)	2.78 (1.82–4.24)	1.50 (0.78–2.88)	0.32 (0.21–0.50)	0.60 (0.37–0.97)	0.95 (0.62–1.44)
Atrial or junctional or ventricular premature beats	0.96 (0.64–1.43)	1.75 (1.24–2.46)	5.74 (3.81–8.66)	1.17 (0.75–1.81)	1.25 (0.89–1.75)	0.79 (0.49–1.27)	0.87 (0.57–1.33)	0.89 (0.54–1.48)	1.11 (0.83–1.49)
Incomplete RBBB	2.04 (1.33–3.15)	1.40 (0.97–2.03)	1.17 (0.65–2.10)	1.67 (1.12–2.49)	0.88 (0.60–1.30)	1.20 (0.68–2.10)	0.80 (0.50–1.29)	1.00 (0.56–1.76)	1.72 (1.26–2.35)
Mobitz Type I AV conduction defect	1.79 (0.74–4.33)	1.75 (0.93–3.28)	3.43 (1.73–6.80)	1.12 (0.63–2.01)	1.92 (1.05–3.50)	1.01 (0.51–2.02)	0.63 (0.30–1.36)	0.39 (0.15–1.03)	0.42 (0.22–0.82)
Short PR interval	0.57 (0.38–0.86)	0.76 (0.44–1.30)	0.42 (0.21–0.85)	1.35 (0.58–3.15)	0.67 (0.38–1.19)	1.00 (0.46–2.20)	0.79 (0.36–1.77)	0.81 (0.41–1.60)	0.84 (0.51–1.41)

Note: Factors simultaneously adjusted to calculate the odds ratios included gender, age classes, smoking history, hypertension, blood glucose classes, BMI classes, hypercholesterolemia and rural/urban areas. The upper limit and the lower limit of the 95% confidence intervals (CIs) were written in the brackets. Normal ECG was used as the reference. The middle age (45–59 years old) group and the older age (≥60 years old) group were compared with the young group (20–44 years old). The underlined odds ratios indicated the significant associations between the factors and the ECG findings. *LBBB* left bundle branch block, *RBBB* right bundle branch block, *IVCD* intravascular conducting delay, *AV* atrial-ventricular

Regarding the factors influencing other ECG abnormal types except arrhythmias, the results of multivariate logistic regression analysis are displayed in Table 5. Older age (at least 60 years old) was positively associated with ST depression and T abnormalities, tall R wave left, left/right atrial hypertrophy, left axis deviation and low voltage. Smoking was positively associated with Q wave abnormalities, tall R wave right and low voltage. Hypertension significantly increased the odds of having ST depression and T abnormalities, Q wave abnormalities, tall R wave left and left axis deviation (Table 5). Overweight and obesity were positively associated with ST depression and T abnormalities and left axis deviation (Table 5 and Supplemental Table 1). Hypercholesterolemia was positively associated with ST depression and T abnormalities (Table 5).

**Table 5:** The odds ratios of the effects of multiple factors on ECG abnormalities except arrhythmias

ECG abnormalities	Gender (male vs female)	45–59 years old	≥60 years old	Smoking	Hypertension	Diabetes	Obesity	Hyper-cholesterolemia	Rural (vs urban)
ST depression and T abnormalities	0.51 (0.44–0.58)	1.78 (1.58–2.00)	2.45 (2.07–2.89)	1.04 (0.89–1.22)	1.92 (1.69–2.18)	1.17 (0.98–1.39)	1.24 (1.05–1.47)	1.28 (1.09–1.51)	0.97 (0.87–1.09)
Q wave abnormalities	1.15 (0.84–1.58)	1.28 (0.88–1.86)	1.49 (0.97–2.28)	1.62 (1.14–2.29)	2.04 (1.48–2.81)	1.04 (0.65–1.66)	1.25 (0.81–1.94)	1.41 (0.87–2.28)	0.84 (0.62–1.14)
Q wave abnormalities plus ischemic ST-T abnormalities	1.38 (0.57–3.34)	1.00 (0.41–2.45)	4.13 (1.60–10.69)	0.99 (0.38–2.59)	1.39 (0.65–2.96)	0.89 (0.32–2.47)	0.86 (0.28–2.63)	1.83 (0.77–4.32)	0.79 (0.39–1.61)
ST elevation	20.03 (8.26–48.62)	0.45 (0.26–0.77)	0.13 (0.06–0.26)	1.60 (0.98–2.62)	0.77 (0.47–1.27)	0.15 (0.07–0.34)	0.51 (0.23–1.12)	0.52 (0.19–1.44)	1.15 (0.75–1.77)
Tall R wave left	1.95 (1.48–2.58)	1.51 (1.21–1.89)	1.85 (1.38–2.47)	1.02 (0.80–1.30)	4.03 (3.25–5.00)	0.68 (0.47–0.99)	0.32 (0.22–0.45)	1.16 (0.83–1.61)	1.03 (0.83–1.30)
Tall R wave right	0.58 (0.23–1.48)	0.33 (0.14–0.78)	1.29 (0.50–3.35)	2.83 (1.16–6.89)	2.02 (0.91–4.49)	0.92 (0.36–2.32)	0.93 (0.29–2.98)	0.46 (0.17–1.22)	0.42 (0.19–0.96)
Left/right atrial hypertrophy	2.72 (1.04–7.08)	3.27 (1.57–6.79)	8.47 (3.73–19.22)	0.75 (0.30–1.88)	0.56 (0.21–1.47)	1.36 (0.33–5.61)	0.33 (0.08–1.30)	2.41 (0.76–7.67)	1.11 (0.50–2.51)
Left axis deviation	1.36 (1.04–1.79)	2.12 (1.63–2.76)	3.38 (2.43–4.69)	1.08 (0.81–1.45)	1.40 (1.09–1.79)	1.13 (0.81–1.56)	2.04 (1.54–2.72)	1.26 (0.95–1.66)	0.69 (0.55–0.87)
Right axis deviation	1.64 (0.98–2.75)	0.82 (0.45–1.50)	0.98 (0.51–1.88)	1.60 (0.93–2.77)	0.80 (0.48–1.32)	0.79 (0.32–1.93)	0.32 (0.15–0.66)	0.72 (0.36–1.44)	0.60 (0.38–0.96)
Low voltage	0.44 (0.26–0.72)	1.72 (1.15–2.58)	3.26 (1.90–5.60)	1.73 (1.00–3.00)	0.45 (0.28–0.75)	1.52 (0.85–2.71)	0.52 (0.24–1.10)	0.63 (0.36–1.09)	0.90 (0.63–1.26)

Note: Factors simultaneously adjusted to calculate the odds ratios included gender, age classes, smoking history, hypertension, blood glucose classes, BMI classes, hypercholesterolemia and rural/urban areas. The upper limit and the lower limit of the 95% confidence intervals (CIs) were written in the brackets. Normal ECG was used as the reference. The middle age (45–59 years old) group and the older age (≥60 years old) group were compared with the young group (20–44 years old). The underlined odds ratios indicated the significant associations between the factors and the ECG findings

The weighted prevalences of major arrhythmias in participants with none, one, two and at least three CVD risk factors were 1.19, 1.76, 1.95 and 2.17% respectively (Table 6). The presence of CVD risk factors significantly increased the odds of obtaining ST depression and T abnormalities, Q wave abnormalities and tall R wave left, after gender and age were adjusted (Table 6). A history of cardiovascular/cerebrovascular diseases significantly increased the odds of having major arrhythmias, atrial fibrillation/flutter, atrial/junctional/ventricular premature beats, ST depression and T abnormalities, Q wave abnormalities, tall R wave left and left axis deviation, with gender and age adjusted (Table 6). The weighted prevalence of major arrhythmias in participants with a history of cardiovascular/cerebrovascular diseases was as high as 5.72%, while the prevalence in those without the history was only 1.61% (Table 6).

**Table 6:** Prevalences of ECG abnormalities with presence of CVD risk factors and history of cardiovascular/cerebrovascular diseases

ECG abnormalities	CVD risk factors (except age and gender)				History of cardiovascular/cerebrovascular diseases	
	None (%)	One (%)	Two (%)	Three or more (%)	Without (%)	With (%)^a^
**Major arrhythmias**	**1.19**	**1.76**	**1.95**	2.17*	**1.61**	5.72***
Atrial fibrillation or flutter	0.23	0.36	0.12	0.67	0.21	2.70***
Complete LBBB	0.06	0.14	0.07	0.34	0.11	0.17
Complete RBBB	0.55	0.65	1.32**	0.78	0.84	1.99
Nonspecific IVCD	0.16	0.25	0.16	0.27	0.21	0.48
Mobitz Type II or III AV conduction defects	0.03	0.06	0.07	0.05	0.05	0.01
Supraventricular or ventricular rhythm/tachycardia	0.05	0.16	0.14	0.05	0.09	0.10
WPW	0.09	0.11	0.05	0.02	0.08	0.20
**Minor arrhythmias**	**9.38**	**11.63**	**8.84**	**10.84**	**9.95**	**9.28**
Sinus bradycardia	2.93	3.49	2.31	2.00	2.84	1.98
Sinus tachycardia	1.53	1.82	1.55	1.72	1.72	0.70
Atrial or junctional or ventricular premature beats	1.63	1.56	1.83	1.04	1.53	2.30**
Incomplete RBBB	0.83	1.36	0.74	1.31	0.97	0.67
Mobitz Type I AV conduction defect	0.29	0.64	0.40	0.39	0.48	0.27
Short PR interval	0.94	0.65	0.49	0.27	0.69	1.64
**Other ECG abnormalities except arrhythmias**						
ST depression and T abnormalities	9.82	10.04***	12.56***	15.36***	10.59	21.83***
Q wave abnormalities	0.94	1.19	1.67**	2.99***	1.24	1.88**
Q wave abnormalities plus ischemic ST-T abnormalities	0.18	0.11	0.11	0.31	0.15	2.16**
ST elevation	0.51	1.78*	0.43	0.73	0.92	0.07
Tall R wave left	2.52	5.45***	6.11***	5.52***	4.18	7.62**
Tall R wave right	0.13	0.19	0.34*	0.12	0.21	0.54
Left/right atrial hypertrophy	0.22	0.39	0.33	0.05	0.33	0.36
Left axis deviation	1.60	2.04*	2.58***	3.99***	2.10	2.95*
Right axis deviation	0.55	0.85	0.45	0.17	0.68	2.01
Low voltage	1.46	0.84	0.49	0.51	0.96	0.75

*LBBB* left bundle branch block, *RBBB* right bundle branch block, *IVCD* intravascular conducting delay, *AV* atrial-ventricular, *CVD* cardiovascular disease

*, ** and *** refer to *p* < 0.05, *p* < 0.01 and *p* < 0.001 respectively. Gender and age were adjusted to calculate the p values by multivariate logistic regression analysis. The prevalences of ECG findings in participants present with CVD risk factors was compared with those absent of CVD risk factors

^a^The prevalences of ECG findings in participants with history of cardiovascular/cerebrovascular diseases was compared with those without history of cardiovascular/cerebrovascular diseases

## Discussion

Previous prospective studies have found that baseline major and minor ECG abnormalities have different levels of impact on the risk of CVD events and all-cause mortality, suggesting that it is crucial to identify and classify baseline ECG abnormalities [4, 5, 10–14]. The prevalences of ECG findings varied in different races. Middle-aged black men had significantly higher prevalence of major ECG abnormalities than middle-aged white men [15, 16]. Non-Hispanic population had significantly higher prevalences of atrial fibrillation, left ventricular hypertrophy and ST depression than Hispanic population [17]. Taiwan Chinese women aged at least 40 years old had higher prevalence of major ECG abnormalities than American White women of about the same age [18]. Currently, no population-based estimation of the prevalence of ECG abnormalities has been reported in China. The ECG data contained in the present study were obtained from a large population in a cross-sectional study. The present data provide overall estimates of the prevalence of ECG findings in Chinese adults (aged ≥20 years old) and the relationships between ECG abnormalities and gender, age and CVD risk factors.

Ethnicity is an important factor affecting the prevalences of ECG findings. We compared our study with previous population-based studies that had similar age range as ours but had different racial background. The prevalences of some arrhythmias in our study were close to the results in a study of American Hispanics/Latinos aged 18–74 years old and another study of Belgians aged 25–74 years old [1, 2]. In these two foreign studies, atrial fibrillation or flutter accounted for 0.30–0.55% in men and 0.04–0.33% in women [1, 2]. In our study, the weighted prevalences of atrial fibrillation or flutter were 0.35 and 0.20% in men and women respectively, which were between the ranges of these two previous studies. Complete RBBB was the most prevalent arrhythmia type in the American Hispanics/Latinos study and the Belgian study, as well as in our study [1, 2]. In our study, complete RBBB accounted for 1.16 and 0.55% in men and women respectively, which were also between the ranges reported in these two previous studies [1, 2]. However, we had higher prevalences of WPW, Mobitz Type II or III AV conduction defects and supraventricular or ventricular rhythm/tachycardia, but lower prevalences of nonspecific IVCD and artificial pacemaker, compared with the American Hispanics/Latinos study [2]. With regard to ST-T abnormalities, because some subcenters in our study recorded the ST depression and T wave abnormalities together and did not differentiate the major and minor ST-T abnormalities, we eventually calculated the prevalence of ST depression (MC 4–1, 4–2, 4–3, 4–4) and T abnormalities (MC 5–1, 5–2, 5–3 or 5–4) together in the population. ST depression and T abnormalities accounted for 7.54 and 14.32% in men and women respectively in our study, while the prevalences of ST depression and T abnormalities were approximately 8 and 10% in men and women respectively in the American Hispanics/Latinos study [2].

The weighted prevalences of several ECG abnormalities increased with age in both men and women in our study, confirming the age-related increase in the prevalence of these ECG abnormalities reported in previous studies [1–3, 19, 20]. Older age (at least 60 years old) increased the likelihood of having atrial fibrillation/flutter by about 8 times, while metabolic and geographic factors had no significant association with atrial fibrillation/flutter in comparison with the reference group (20–44 years old) (Table 4). Older age not only increased the prevalence of atrial fibrillation/flutter in cross-sectional studies, but also increased the incidence of atrial fibrillation/flutter in retrospective and prospective studies, suggesting that older age had tremendous association with atrial fibrillation/flutter [1, 21, 22]. The likelihoods of having complete LBBB and complete RBBB in the 60 years old or older group were both more than 4 times higher than those in the reference group (20–44 years old) in our study (Table 4). A Korean cross-sectional study also found that the prevalences of complete LBBB and complete RBBB both increased with age [23]. In addition, older age also significantly increased the prevalences of ST depression and T abnormalities, tall R wave left and left/right atrial hypertrophy. These results suggest that it is essential to conduct ECG regularly for people at lease 60 years old to screen ECG abnormalities especially arrhythmias.

Among the ECG abnormalities, atrial fibrillation/flutter was broadly studied before and recently. Atrial fibrillation/flutter, well known as a critical risk factor for stroke, was also indicated as an independent risk factor for ventricular fibrillation in a population-based case-control study [24]. In a cross-sectional study of participants aged at least 35 years old in China, the prevalences of atrial fibrillation were 0.78 and 0.76% in men and women, respectively [7]. The inclusion of a history of atrial fibrillation in addition to ECG records and the older age of the participants could have contributed to the higher prevalence of atrial fibrillation observed in this previous study than in our study [7]. The factors associated with atrial fibrillation/flutter were mainly older age and the history of cardiovascular/cerebrovascular diseases (Tables 4 and 6). However, in contrast to previously reported studies, we found that smoking history, hypertension, diabetes, obesity and hypercholesterolemia were not significantly related with atrial fibrillation [7, 25]. The prevalence of having atrial fibrillation/flutter in participants with a history of cardiovascular/cerebrovascular diseases was as high as 2.7%, which were much higher than the prevalence in those without the history. The prevalence of atrial fibrillation varied from 0.6 to 4.1% in patients with cardiovascular diseases in previous studies, while the prevalence of cardiovascular diseases was as high as 38.2% in patients with atrial fibrillation [26–28]. Atrial fibrillation had close association with cerebrovascular diseases especially stroke [29, 30]. The famous Framingham Study indicated that there was a nearly fivefold excess of stroke in the subjects with atrial fibrillation compared with those free of atrial fibrillation after 34 years of follow-up [30]. Therefore, it is important to screen atrial fibrillation/flutter in patients with cardiovascular/cerebrovascular diseases.

The odds of having ischemic ST-T abnormalities significantly increased with hypertension, obesity and hypercholesterolemia, confirming the association between metabolic factors and ischemic ECG abnormalities [1, 2]. Metabolic factors, including hypertension, obesity and hypercholesterolemia, significantly increased the risk of having ischemic heart diseases in prospective studies [31, 32]. It is important to identify and control these metabolic factors in order to prevent ischemic heart diseases. High R wave left is a main ECG manifestation of left ventricular hypertrophy. Our study and previous studies all presented that hypertension was a key associated factor for left ventricular hypertrophy or high R wave left [33, 34]. Another previous study followed up hypertensive patients for a mean time of approximately 10 years and found that left ventricular hypertrophy significantly increased the risk of CVD events [35]. Thus, it is crucial to identify left ventricular hypertrophy in hypertensive patients, while ECG provides a convenient way to identify it. Meanwhile, blood pressure control in hypertensive patients can prevent the development of left ventricular hypertrophy [36].

Our study had some limitations. First, the original design of the ECG recording didn’t include PR, QRS, QT and QTc interval durations and heart rates. Some subcenters recorded the ST depression and T wave abnormalities together; thus, the major ST-T abnormalities could not be differentiated from the minor ST-T abnormalities. Second, there was some observer bias due to different observers in different subcenters. However, in each subcenter, two well-trained physicians read the ECG, checked with each other, and then recorded the ECG results. If they could not reach a consensus about the ECG result, they would consult the ECG specialists in the Project Committee. The consensus of two well-trained physicians on each ECG might reduce some inter-observer and intra-observer bias. Third, we didn’t follow up these participants to investigate the progression of ECG findings and metabolic characteristics. In addition, there were about 24% participants excluded from the ECG analysis mainly due to the lack of ECG data. However, we analyzed the characteristics of the participants included and those excluded in this article (Supplemental Table 2). The proportions of the two genders and the proportions of the six age classes, which were the two most important demographic characteristics, showed no significant differences between the included participants and the excluded participants.

## Conclusions

Our study provides estimates of the prevalences of ECG findings in Chinese adults. Gender, age, CVD risk factors and history of cardiovascular/cerebrovascular diseases were significantly associated with ECG abnormalities.

## Supplementary information

**Additional file 1: Table S1.** The odds ratios of the effects of prediabetes and overweight on ECG abnormalities. **Table S2.** Comparison of the characteristics of the included and excluded populations

## Data Availability

The datasets used and/or analyzed in the current study are available from the corresponding author upon reasonable request.

## Abbreviations

ECG: Electrocardiogram
MC: Minnesota coding
CVD: Cardiovascular diseases
LBBB: Left bundle branch block
RBBB: Right bundle branch block
IVCD: Intraventricular conduction delay
AV: Atrial-ventricular
WPW: Wolff-Parkinson-white
CHD: Coronary heart disease
