# Supplementary material for: Prevalences and associated factors of electrocardiographic abnormalities in Chinese adults: a cross-sectional study
Source: BMC Cardiovasc Disord. 2020 Sep 11;20:414. doi: 10.1186/s12872-020-01698-5 (PMC7488680; doi:10.1186/s12872-020-01698-5)
Supplement: Supplementary file 1 — Additional file 1: Table S1. The odds ratios of the effects of prediabetes and overweight on ECG abnormalities. Table S2. Comparison of the characteristics of the included and excluded populations [file 12872_2020_1698_MOESM1_ESM.docx]

Supplemental table 1. The odds ratios of the effects of prediabetes and overweight on ECG abnormalities

| ECG abnormalities | Pre-diabetes | Overweight |
| --- | --- | --- |
| **Major arrhythmias** | **1.40 (0.99-1.96)** | **1.13 (0.82-1.55)** |
| Atrial fibrillation/flutter | 1.42 (0.59-3.45) | 1.35 (0.61-3.01) |
| Complete LBBB | 1.66 (0.47-5.83) | 0.95 (0.28-3.26) |
| Complete RBBB | 1.32 (0.81-2.17) | 1.10 (0.69-1.76) |
| Nonspecific IVCD | 1.69 (0.67-4.31) | 1.33 (0.56-3.15) |
| Mobitz Type II or III AV conduction defects | 6.40 (1.69-24.20) | 0.75 (0.10-5.43) |
| Supraventricular or ventricular rhythm/tachycardia | 0.98 (0.37-2.58) | 0.85 (0.26-2.73) |
| WPW | 0.29 (0.04-2.22) | 1.63 (0.69-3.89) |
| **Minor arrhythmias** | **1.10 (0.89-1.34)** | **0.76 (0.63-0.91)** |
| Sinus bradycardia | 0.77 (0.53-1.13) | 0.77 (0.60-0.98) |
| Sinus tachycardia | 1.64 (0.89-3.05) | 0.60 (0.31-1.16) |
| Atrial or junctional or ventricular premature beats | 1.02 (0.67-1.54) | 0.90 (0.64-1.27) |
| Incomplete RBBB | 1.30 (0.81-2.09) | 0.69 (0.47-1.00) |
| Mobitz Type I AV conduction defect | 1.68 (0.68-4.13) | 1.05 (0.53-2.06) |
| Short PR interval | 0.76 (0.42-1.35) | 1.24 (0.60-2.55) |
| **Other ECG abnormalities** |  |  |
| ST depression and T abnormalities | 1.32 (1.12-1.56) | 1.20 (1.05-1.37) |
| Q wave abnormalities | 0.75 (0.48-1.18) | 1.01 (0.74-1.39) |
| Q wave abnormalities plus ischemic ST-T abnormalities | 1.69 (0.70-4.09) | 1.35 (0.60-3.04) |
| ST elevation | 1.19 (0.52-2.72) | 0.78 (0.49-1.25) |
| Tall R wave left | 1.32 (0.95-1.83) | 0.65 (0.50-0.83) |
| Tall R wave right | 0.94 (0.26-3.38) | 1.60 (0.58-4.41) |
| Left/right atrial hypertrophy | 1.27 (0.60-2.73) | 0.38 (0.15-0.95) |
| Left axis deviation | 1.30 (0.98-1.73) | 1.51 (1.17-1.95) |
| Right axis deviation | 0.72 (0.31-1.64) | 0.58 (0.36-0.93) |
| Low voltage | 1.02 (0.59-1.76) | 0.54 (0.36-0.81) |

Note: Factors simultaneously adjusted to calculate the odds ratios included gender, age classes, smoking history, hypertension, blood glucose classes, BMI classes, hypercholesterolemia and rural/urban areas. The upper limit and the lower limit of the 95% confidence intervals (CIs) were written in the brackets. Normal ECG was used as the reference. The underlined odds ratios indicated the significant associations between the factors and the ECG findings. LBBB, left bundle branch block; RBBB, right bundle branch block; IVCD, intravascular conducting delay; AV, atrial-ventricular.

Supplemental table 2. Comparison of the characteristics of the included and excluded populations

| Characteristics | Included  (n = 34, 965) | Excluded  (n = 11, 274) | P values |
| --- | --- | --- | --- |
| Gender |  |  | 0.938 |
| Male, % | 49.4 | 49.5 |  |
| Female, % | 50.6 | 50.5 |  |
| Age, y [mean (95% CI)] | 44.8 (44.7, 44.8) | 45.0 (44.8, 45.1) | 0.035 |
| Age classes | | | 0.796 |
| 20-29 years old, % | 17.4 | 17.6 | 0.922 |
| 30-39 years old, % | 23.9 | 24.1 | 0.892 |
| 40-49 years old, % | 22.4 | 21.4 | 0.389 |
| 50-59 years old, % | 18.2 | 18.2 | 0.986 |
| 60-69 years old, % | 10.1 | 10.5 | 0.600 |
| ≥70 years old, % | 7.9 | 8.1 | 0.803 |
| BMI, kg/m^2^ [mean (95% CI)] | 23.8 (23.7, 23.8) | 23.3 (23.1, 23.5) | <0.001 |
| Overweight, % | 32.0 | 26.9 | <0.001 |
| Obesity, % | 12.4 | 10.9 | 0.063 |
| Smoking, % | 30.6 | 29.8 | 0.540 |
| Hypertension, % | 28.3 | 22.0 | <0.001 |
| Prediabetes, % | 15.8 | 13.6 | 0.015 |
| Diabetes, % | 9.9 | 8.3 | 0.011 |
| Hypercholesterolemia, % | 10.8 | 10.7 | 0.942 |

Among the original 47,325 participants, 1,086 persons missed demographic information or glucose level data. The database didn’t include the data of the 1086 persons. Thus, we compared the characteristics between the 11, 274 excluded persons and the 34, 965 included persons. The prevalence calculation and significance evaluation, performed using SUDAAN software (version 10, Research Triangle Institute) in this study, were weighted to represent the population of Chinese adults (≥20 years old) based on the Chinese population distribution data in 2006. The percentages shown above were compared by Chi-square test. The quantitative values of age and BMI were indicated as mean (95% CI) and compared by two-tailed t test. BMI, body mass index; FPG, fasting plasma glucose level; PG2h, plasma glucose level of 2 hours after oral glucose tolerance test; CI, confidence interval.
